# Os Efeitos da Doxorrubicina na Biossíntese e no Metabolismo do Heme em Cardiomiócitos

**DOI:** 10.36660/abc.20190437

**Published:** 2021-02-19

**Authors:** Zuoyan Wang, Junyi Gao, Haobo Teng, Jianjun Peng

**Affiliations:** 1 Capital Medical UniversityBeijing Shijitan HospitalBeijingChinaCapital Medical University Affiliated Beijing Shijitan Hospital,Beijing - China

**Keywords:** Doxorrubicina, Biosíntese, Heme, Miócitos Cardíacos, Cardiotoxicidade, ALAS

## Abstract

**Fundamento:**

A doxorrubicina está associada à cardiotoxicidade e à morbidade cardíaca tardia. O heme está relacionado ao stress oxidativo celular. Entretanto, sua regulação específica em cardiomiócitos sob os efeitos de doxorrubicina ainda não foi documentada.

**Objetivo:**

Nosso objetivo é avaliar as alterações de enzimas limitantes de velocidade no caminho metabólico do heme sob o efeito de doxorrubicina.

**Métodos:**

Cardiomiócitos H9c2 com doxorrubicina em concentrações diferentes (1, 2, 5, 10μM respectivamente). Os testes de PCR em tempo real e Western Blot foram usados para determinar a expressão de proteína e mRNA para quatro enzimas cruciais (ALAS1, ALAS2, HOX-1, e HOX-2) que regulam o metabolismo do heme celular, e os níveis de heme foram detectados por ELISA. Um *p*<0,01 foi considerado significativo.

**Resultados:**

Observamos um padrão com alteração dependendo da dose nos níveis de heme nas células H9c2 com o nível mais alto na concentração de 5μM de doxorrubicina, o que ocorreu sincronicamente com o nível mais alto de regulação para cima de ALAS1, bem como as enzimas degenerativas HOX-1 e HOX-2 na expressão de proteína e mRNA. Em contraste, observamos que a ALAS2 foi regulada para baixo gradualmente, inversamente proporcional às concentrações de doxorrubicina.

**Conclusão:**

O aumento da expressão de ALAS1 pode ter um papel na elevação do nível do heme quando o cardiomiócito H9c2 for exposto à doxorrubicina, e pode ser um alvo terapêutico para a toxicidade miocárdica induzida por doxorrubicina. (Arq Bras Cardiol. 2021; 116(2):315-322)

## Introdução

Com a progressão contínua das drogas antitumorais e radioterapia, o índice de sobrevivência dos pacientes com tumores malignos melhorou, e seu período de sobrevivência é significativamente aumentado. Entretanto, o uso disseminado de drogas antitumorais é acompanhado de um aumento dos eventos cardiovasculares adversos, que afeta a sobrevivência e a qualidade de vida do paciente. Antraciclinas são drogas derivadas da estreptomicina, incluindo a doxorrubicina, e da epirrubicina, que são amplamente utilizadas para tratar câncer de mama, câncer do pulmão de pequenas células, mieloma, sarcoma, linfoma e leucemia. A miocardiopatia e a insuficiência cardíaca subsequente são as manifestações mais graves de cardiotoxicidade causadas por drogas antraciclinas na quimioterapia. Além disso, a toxicidade cardiovascular das antraciclinas depende da dose e é irreversível.^1,2^

O mecanismo exato da toxicidade miocárdica induzida por drogas antraciclinas ainda não é clara, embora várias teorias tenham sido propostas, incluindo a inibição da replicação de DNA e transcrição de RNA; o dano ao DNA causado por radicais livres, peroxidação lipídica e alquilação; a ligação cruzada do DNA; interferência no desenrolamento do DNA; a inibição da topoisomerase II, etc.

O heme é uma porfirina ligante importante, como suplemento da proteína do heme, tem a função fisiológica de transportar o O_2_ no corpo. Muitas funções biológicas relacionadas à vida, tais como o transporte de elétrons, o armazenamento de oxigênio, a transdução de sinal, e a expressão genética são controladas pelas várias proteínas heme. Estudos recentes detectaram que os níveis de heme aumentaram significativamente em modelos de insuficiência cardíaca isquêmica miocárdica de ratos, sugerindo que o heme possa ter um papel importante em lesões miocárdicas isquêmicas e hipóxicas.^3,4^ Entretanto, não há trabalhos publicados na literatura que exploram a alteração da biossíntese ou do metabolismo do heme em cardiomiócitos, sob tratamento com antraciclinas.

A biossíntese do heme é iniciada pela formação do ácido δ-aminolevulínico (ALA) a partir da glicina (Gly) e succinil coenzima A, catalisada pela sintase do ALA (ALAS), que tem duas isoenzimas: ALAS1 e ALAS2. Por outro lado, a heme oxigenase (HOX) media o primeiro passo do catabolismo do heme, e divide o heme para formar a biliverdina. Duas isoformas ativas de HOX foram identificadas, a HOX-1 induzível, e a menos regulada HOX-2.

O objetivo deste estudo foi elucidar as alterações de perfis de quatro enzimas cruciais, a sintase do ácido aminolevulínico 1 (ALAS1), a sintase do ácido aminolevulínico 2 (ALAS2), heme oxigenase 1 (HOX-1), e heme oxigenase 2 (HOX-2), no cardiomiócito H9c2 em tratamento com doxorrubicina.

## Métodos

### Cultura celular e tratamento com doxorrubicina

Foram comprados cardiomiócitos H9c2 da American Type Culture Collection (ATCC, Manassas, VA, EUA) e foi feita a cultura em meio DMEM/F12 contendo 10% de soro fetal bovino e 1% de meio de anticorpo duplo, nas condições de 37 °C e 5% de CO2, em câmara umidificada. As células de H9c2 foram distribuídas em placas de titulação de 6 poços, num índice de 2 × 10^5^ células/poço em um volume de 2 mL e em cultura de meio DMEM/F12 contendo 10% de soro fetal bovino, por 24 h. Depois disso, doses diferentes de doxorrubicina (1,2,5,10 μM, respectivamente) foram adicionados aos poços da placa de titulação. Consideramos células não tratadas com doxorrubicina (tratadas com solução salina) como grupo de controle. Depois de 24 horas de incubação a 37ºC, as células de poços diferentes foram coletadas separadamente por centrifugação (10000 rpm por 10 minutos a 4°C) e usadas para estudos posteriores abaixo.

### PCR quantitativo em tempo real

O RNA total foi extraído das células H9c2 de todos os grupos utilizando o TRIzol (Invitrogen; Thermo Fisher Scientific, Inc., Waltham, MA, EUA), e foi feita a transcrição reversa com o kit SuperScript Double-Strand Synthesis (Invitrogen; Thermo Fisher Scientific, Inc., Waltham, MA, USA) de acordo com as instruções do fabricante, e amplificado em um sistema de PCR de tempo real 7500 Fast com SYBR GreenER qPCR SuperMix Universal (Invitrogen; Thermo Fisher Scientific, Inc.). Os primers usados para amplificar ALAS1, ALAS2, HOX-1, e HOX-2 foram projetados usando os softwares Primer3 (v. 0.4.0). Os seguintes primers foram usados para o PCR em tempo real:

ALAS1-F:TTGCCAAAGTCCGTTTCC

R: TGTAGTCATCTGCCATAGGG 3’;

ALAS2-F: TCAAGGGAGAGGAGGGTCAAG

R: ACGAGGCACAGTTGGGTAG

HOX-1-F:TCGACAACCCCACCAAGTT

R:CTGGCGAAGAAACTCTGTCT

HOX-2-F:GCTTACACTCGTTACATGGG

R: CACATGCTCGAACAGGTAGA

GAPDH-F: GATGACATCAAGAAGGTGGTGA

R:ACCCTGTTGCTGTAGCCATATTC.

As condições de reação foram as seguintes: 95 °C por 3 min; 95 °C por 30 seg, 55 °C por 20 seg, 72 °C por 20 seg com 40 ciclos. Foram realizadas análises de curva de fusão para verificar a especificidade de sua amplificação. Os valores de expressão de todos os genes-alvo de cada amostra foram calculados normalizando com GAPDH controle interno, e calculados utilizando o método 2-ΔΔCT.

### Western Blot

Células H9c2 foram centrifugadas por tripsinização por 5 min a 1.000 x g e lavadas com PBS frio. Em seguida, as células H9c2 foram ressuspensas em tampão de lise com inibidores de protease e fosfatase. O lisado celular foi mantido em gelo e agitado no vórtex. Depois da centrifugação por 20 a 13,000×g, o sobrenadante foi separado e armazenado a −80 °C até o uso. Depois da desnaturação, 20μg de proteína total foram colocados em eletroforese em gel de acrilamida contendo dodecil sulfato de sódio 12% (SDS-PAGE), e foram transferidos por eletroforese para uma membrana de fluoreto de polivinilideno (PVDF). Depois do bloqueio com TBST (50mM Tris-HCl, pH 8;154 mM NaCl e 0,1% tween20) por 2 horas em temperatura ambiente, as membranas foram testadas com anticorpos específicos em relação a ALAS1 (diluição,1:1.000; policlonal de coelho; nº cat. 16200-1-AP; Proteintech), ALAS2 (diluição,1:1.000; policlonal de coelho; nº cat. ab136799; Abcam), HOX-1 (diluição,1:1.000; policlonal de coelho; nº cat. ab230513; Abcam), HOX-2(diluição,1:500; policlonal de coelho; nº cat. ab229960; Abcam) e GAPDH (diluição, 1:1.000; clone 6C5; cat. no. ab8245; Abcam) durante a noite a 4°C. Anticorpos secundários titulados com Horseradish peroxidase (HRP) (1:1.000 diluição; nº cat. A0208, A0216; Beyotime) foram acrescentados, após a incubação em temperatura ambiente por 2 horas. As membranas foram visualizadas com kits de quimioluminescência amplificada (Biorbyt, Ltd., Cambridge, Reino Unido). Para quantificar a expressão de proteína direcionada, os filmes de raio X foram escaneados, e as intensidades de banda foram quantificadas pelo software ImageJ 1.47. Os resultados foram normalizados em GAPDH.

### Medição de heme intracelular

O ensaio de imunoabsorção enzimática (ELISA) foi utilizado para determinar os níveis de heme intracelular. As células H9c2 de cada grupo foram lisadas e submetidas a centrifugação a 12000rpm por 15 minutos a 4 °C para retirar detritos. A concentração de proteína do lisado celular foi quantificada pelo kit de Ensaio de Proteína BCA (Beyotime). O heme nos lisados celulares de H9c2 foi medido utilizando-se o Ensaio Heme QuantiChrom (BioAssay Systems), seguindo o protocolo do fabricante e normalizado a uma concentração de proteína de cada amostra.

### Citometria de fluxo para determinar o índice de apoptose em células H9c2

A apoptose foi avaliada utilizando-se o kit Annexin V-FITC/PI Apoptosis Detection kit (KeyGEN, China) de acordo com o protocolo do fabricante. Depois da intervenção, aproximadamente 1×10^5^ células H9c2 de cada grupo foram lavadas, digeridas com tripsina e as células ressuspensas com 1x PBS (4°C), centrifugadas a 2.000 rpm por 5-10 minutos para lavar as células. As células foram ressuspensas em 500μl do tampão, seguido da adição de 5 μl de Annexin V-FITC e 5 μl de PI. As células foram incubadas no escuro por 15 minutos em temperatura ambiente. Em seguida, as células de cada grupo foram examinadas quanto ao índice de apoptose por citometria de fluxo (BD Accuri™ C6), e o experimento foi repetido três vezes. O índice de apoptose foi quantificado como a porcentagem de células coradas com Annexin V.

### Análise estatística

Os dados no presente estudo são normalmente distribuídos, o que foi verificado pelo teste de Shapiro-Wilk, e são expressos como média ± SD. A ANOVA de fator único, seguida do teste post hoc de Tukey, foi usada para examinar a significância estatística das diferenças entre os grupos. A análise estatística foi realizada utilizando-se o software SPSS 24.0 Statistical Package Program for Windows (SPSS Inc., Chicago, IL, EUA). Um p-valor bilateral de <0,01 foi considerado estatisticamente significativo.

## Resultados

### Alterações nos níveis de heme em células H9c2 com concentrações diferentes de doxorrubicina

**Figura 1:**
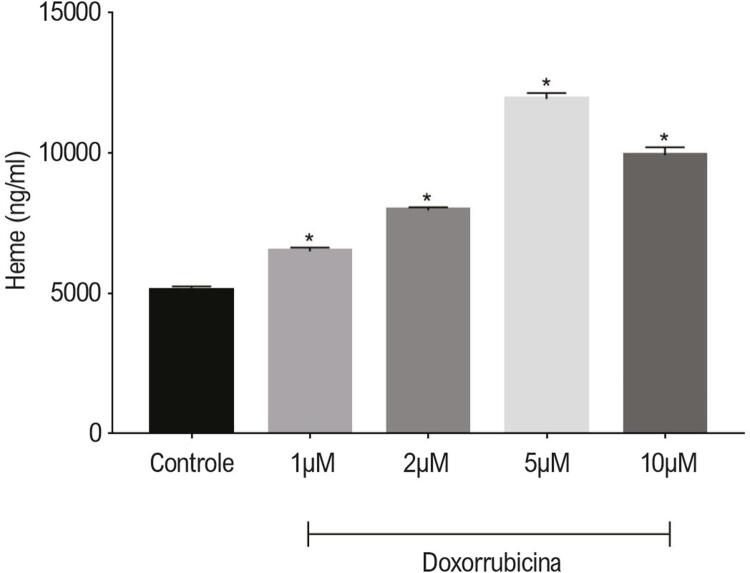
– Efeitos da doxorrubicina nos níveis de heme nas células H9c2 exposta a solução salina (grupo de controle) ou doxorrubicina com concentração diferente por 24 horas. Níveis de heme medidos por ELISA. Os dados são apresentados como média±desvio padrão. *p<0,01, em comparação ao grupo de controle.

Conforme mostrado na Figura 1, em comparação com o grupo de controle (5088,4±153,1ng/ml), os níveis de heme das células H9c2 foram significativamente regulados para cima 1,27 vezes (6493,1±138,8 ng/ml), 1,56 vezes (7498,9±110,2ng/ml), e no máximo um aumento de 2,34 vezes (11896,6±187,3ng/ml) nos grupos com 1μM, 2μM, e 5μM de doxorrubicina, separadamente (p<0,01). Essa tendência à regulação para cima passou a ter um aumento de 1,95 vezes (9911,9±286,8ng/ml) no grupo com 10μM de doxorrubicina, em comparação com grupo de controle (p<0,01).

### Efeitos da doxorrubicina nos índices de apoptose de células H9c2

**Figura 2:**
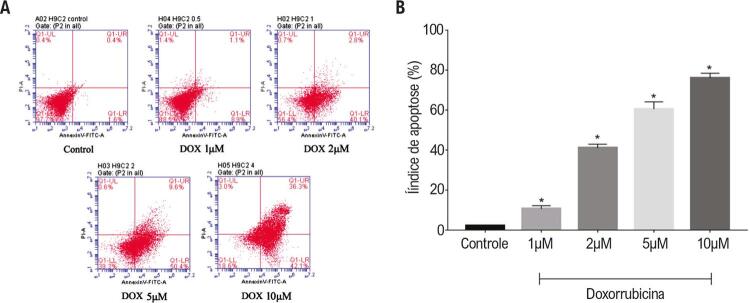
– Análise por citometria de fluxo dos efeitos da doxorrubicina na viabilidade das células H9c2 As células H9c2 foram pré-tratadas com solução salina (controle) e doxorrubicina a 1,2,5 e 10μM respectivamente por 24h. (A) Análises representativas de citometria de fluxo de cinco experimentos separados correspondentes ao controle a às várias concentrações de tratamento com doxorrubicina, respectivamente. (B) Gráfico estatístico de coloração annexin V-FITC/PI. Resultados expressos como média±desvio padrão. *p<0,01, em comparação ao grupo de controle.

A análise de citometria de fluxo demonstrou que, em comparação com o grupo de controle tratado com solução salina, o índice de apoptose de células H9c2 tratadas com concentrações diferentes de doxorrubicina aumentou significativamente, conforme mostrado na Figura 2. Quando incubado com 1,2,5, e 10μM de doxorrubicina por 24 horas, o índice total de apoptose, incluindo a apoptose de estágio inicial e final, de H9c2 foi aumentado para 10,6±1,6%, 41,1±1,9%, 60,5±3,6%, e 76,0±2,5% respectivamente, em comparação com 2,1±0,5% no grupo de controle (p<0,01).

Regulação da sintase do ácido aminolevulínico 1 (ALAS1) e da sintase do ácido aminolevulínico 2 (ALAS2) expressão de RNA mensageiro (mRNA) em células H9c2 depois do tratamento com doxorrubicina

**Figura 3:**
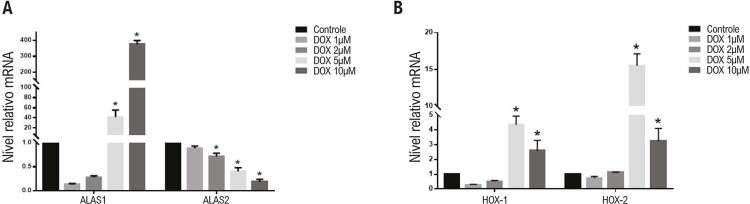
– (A) alteração da expressão de mRNA de ALAS1 e ALAS2 em tratamento com doxorrubicina. sintase do ácido aminolevulínico 1; ALAS2, sintase do ácido aminolevulínico 2. (B) Características da alteração da expressão de mRNA para enzimas catabólicas do heme em tratamento com doxorrubicina. HOX-1, heme oxigenase 1; HOX-2, heme oxigenase2. * indica p<0,01 versus grupo de controle.

Como primeiro passo, foram avaliadas as enzimas limitantes de velocidade da síntese do heme que ocorre nas mitocôndrias, a expressão do mRNA da ALAS1 e ALAS2 em tratamento com doxorrubicina. Depois da incubação com 1μM e 2μM doxorrubicina, as expressões de mRNA no ALAS1 foram reguladas para baixo (embora não sejam estatisticamente significativos). Depois de tratar com 5μM e 10μM de doxorrubicina, as expressões de mRNA de ALAS1 foram estatisticamente aumentadas até 41,1 vezes 375,3 vezes, em comparação com o grupo de controle separadamente. Diferente da ALAS1, a expressão de mRNA de ALAS2 demonstrou um uma regulação para baixo significativa (grupo 1μM:0,88 vezes, grupo 2μM: 0,83vezes, grupo de 5μM: 0,49 vezes, grupo 10μM:0,31 vezes respectivamente, conforme mostrado na Figura 3A.)

### Regulação da expressão do mRNA para as HOX-1 e HOX-2 em células H9c2 após o tratamento com doxorrubicina

Foram examinadas as enzimas citoplasmáticas limitantes de velocidade in catabolismo do heme, HOX-1, e HOX-2. Detectamos que, depois de incubado com um nível crescente de doxorrubicina, o mRNA de HOX-1 e HOX-2 exibiu o mesmo padrão de regulação, embora com níveis de alteração diferenciados. Não houve alteração significativa na expressão do mRNA depois do tratamento com 1μM e 2μM, comparado com o grupo de controle, nem em HOX-1, nem HOX-2. Depois de incubado com 5μM de doxorrubicina, a expressão de mRNA para HOX-1 e HOX-2 se regularam para uma mudança para cima de 4,3 vezes e 15,5 vezes, respectivamente (p<0,01). Entretanto, após o tratamento com 10μM de doxorrubicina, a regulação para cima diminuiu até o nível de 2,6 vezes e 3,2 vezes separadamente, em comparação com o grupo de controle, (p<0,01), conforme mostrado na Figura 3B

### Regulação dos níveis de proteína de ALAS1, ALAS2, HOX-1, e HOX-2 após o tratamento com doxorrubicina

**Figura 4:**
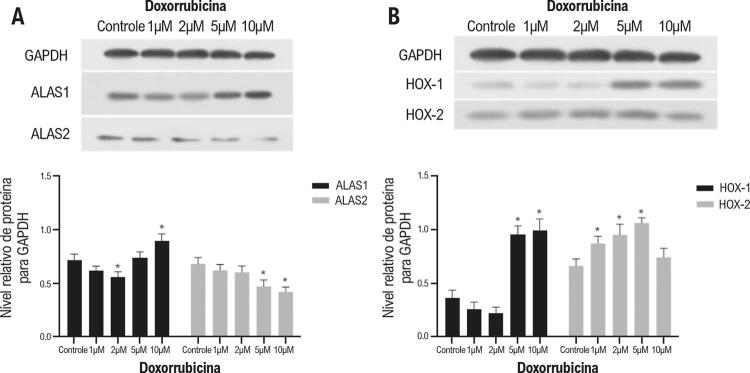
– Análise de dados de Western Blot para alterações de níveis de proteína ALAS (A) e HOX (B) em células H9c2 em concentrações de doxorrubicina diferentes. ALAS1: sintase do ácido aminolevulínico 1; ALAS2: sintase do ácido aminolevulínico; HOX-1: heme oxigenase 1; HOX-2: heme oxigenase 2. Cada teste Western Blot foi realizado três vezes. * indica p<0,01 versus grupo de controle.

A análise de Western Blot demonstrou que, de forma idêntica ao padrão de expressão de mRNA, os níveis de proteína ALAS1 foram regulados para baixo significativamente no grupo de 2μM de doxorrubicina, e regulado para cima no grupo de 10μM de doxorrubicina, em comparação com o grupo de controle. Observou-se que os níveis de proteína foram regulados para baixo progressivamente, à medida que a concentração de doxorrubicina aumentou de 1μM a 10μM, conforme mostrado na Figura 4A.

A expressão de proteína de HOX-1 foi ligeiramente regulada para baixo no tratamento com 1μM e 2μM de doxorrubicina, e depois regulada para cima de forma abrupta, em comparação com o grupo de controle. No entanto, de forma inconsistente com o padrão de expressão de mRNA, detectamos um aumento progressivo no nível de proteína HOX-2 com o aumento da concentração de doxorrubicina de 1μM a 5μM, e, em seguida, voltou para o nível de linha de base quando tratado com 10μM de doxorrubicina (Figura 4B).

## Discussão

Doxorrubicina é um tipo de droga antraciclina que é uma droga antitumoral eficiente de amplo espectro. Ela é amplamente utilizada no tratamento de vários tumores malignos, tais como câncer de mama, câncer de pulmão e linfoma.^5^ Entretanto, os efeitos cardíacos tóxicos da doxorrubicina na dose clínica para quimioterapia são graves e dependem da dose, o que pode levar a cardiomiopatia e insuficiência cardíaca congestiva, e, portanto, limitam muito seu uso clínico.^6^ Diferente do mecanismo de sua atividade antitumoral, o mecanismo primário de toxicidade cardíaca induzido pela doxorrubicina, é a geração de espécies reativas do oxigênio (ERO) e a promoção subsequente do stress oxidativo miocárdico.^7^ O heme é um mediador essencial da disponibilidade bioquímica do ferro.^8^ A função da molécula do heme varia de acordo com a proteína ligante com a qual é coordenado. Ele pode funcionar como mediador de transporte e armazenamento de oxigênio em hemoglobina^9^ ou mioglobina,^10^ e, por outro lado, ele age como um transportador de elétrons em citocromos, e é a fonte crítica de ferro redox-ativo.^11^ Bhoite-Solomon et al. identificaram que o heme livre é tóxico para o miocárdio, e causa citólise por meio de dano no sarcolema, dependendo da concentração.^12^ Além dos cardiomiócitos, o heme livre também pode ser tóxico às células epiteliais humanas e a células semelhantes a neurônios,^13,14^por meio do stress oxidativo causado pela apoptose ou necrose da célula. Além disso, o heme livre pode causar dano celular endotelial, ao estimular a expressão de fatores inflamatórios.^15,16^

Entretanto, diferentemente dos vários estudos sobre dano oxidativo a cardiomiócitos mediados por ferro tratados com drogas antraciclinas, a variação do nível de heme intracelular e a regulação dos processos de sua síntese e metabolismo não foram bem avaliados no cardiomiócito em tratamento com antraciclina. No presente estudo, examinamos sistematicamente as características de variação de enzimas biossintéticas e degradadoras do heme pela primeira vez.

No caminho de biossíntese, há oito enzimas envolvidas, dentre as quais quatro são enzimas mitocondriais, e quatro são enzimas citoplasmáticas.^17^ Na primeira etapa da síntese do heme, a glicina e succinil coenzima A são condensadas no ácido δ-aminolevulínico (ALA). Essa reação precisa ser catalisada pela sintase do ácido aminolevulínico (ALAS).^18^ Há dois tipos de ALAS: ALAS1 e ALAS2. A primeira tem uma expressão universal, enquanto a última é predominantemente expressa em células precursoras de hemácias.^19^ No presente estudo, observamos que não apenas a ALAS1, mas também a ALAS2 foram expressas nas células H9c2 em níveis de linha de base. Após o tratamento com doxorrubicina, a expressão de mRNA de ALAS1 e ALAS2 apresentam dois padrões de alteração distintos. A expressão de proteína e mRNA de ALAS1, a princípio, apresentou uma tendência à inibição (embora não fosse estatisticamente significativa em nosso estudo) com 1μM e 2μM de doxorrubicina, e, depois, padrões dramáticos de regulação para cima com o tratamento com 5μM e 10μM de doxorrubicina.

Por outro lado, a expressão de proteína e mRNA de ALAS2 foi suprimida gradativamente até o nível mais baixo, quando tratada com 10μM de doxorrubicina, com 69,0% e 35,0% de redução, respectivamente. Esse fenômeno indica que, sob o efeito da doxorrubicina, a regulação de ALAS1 e ALAS2 pode tomar caminhos diferentes e ter objetivos de bioprocessos direcionadores diferentes. A regulação para baixo de ALAS2 pode ser explicada pelo feedback negativo de uma elevação progressiva do nível de heme. Esse efeito pode ser alcançado reprimindo-se a transcrição de mRNA de ALAS2, conforme mostrado em nossos resultados de PCR em tempo real, bem como interrompendo-se a formação de ALAS2 por meio de um bloco do precursor de ALAS2 até as mitocôndrias.^20,21^ Nas hemácias, a expressão de ALAS2 é determinada pela transativação de locais de fatores nucleares GATA-1, CACC box, e NF-E2-ligante na área promotora, e sua síntese é regulada pela quantidade de ferro livre.^22^ Entretanto, os mecanismos de regulação de ALAS2 em cardiomiócitos em tratamento com doxorrubicina precisa ser pesquisada mais a fundo.

Em nosso estudo, ALAS1, que deveria estar estritamente em controle de feedback negativo em nível baixo para evitar o alto nível citotóxico de heme, foi expresso excessivamente nos aspectos do gene e da proteína, apesar do alto nível de heme que existe simultaneamente. Podvinec et al.,^23^ relataram que as drogas que induzem citocromos P450 e outras enzimas associadas que metabolizam drogas podem fazer a regulação para cima de ALAS1 simultaneamente e por meio de transcrição, que foi o primeiro passo na síntese de heme, para coordenar com a necessidade de atividade de citocromo P450 com regulação para cima. Esse processo foi mediado por dois elementos aperfeiçoadores, localizados a 20 e 16 kb a montante do local de início transcricional de ALAS1, e sua interação com os xeno-receptores NR1I2 e NR1I3.^23^ Zordoky et al.,^24^ revelaram que a doxorrubicina causa indução significativa de vários genes P450 do citocromo, tais como expressão genética de CYP1A1, CYP1A2, CYP1B1, e CYP2B2 dependendo da concentração. NR1I2 e NR1I3 também demonstraram ser os receptores nucleares cruciais que mediaram a interação entre a doxorrubicina e as enzimas metabolizantes.^25^ Os mecanismos precisos de regulação de padrões de expressão de ALAS no cardiomiócito precisará ser elucidado em estudos futuros.

A heme oxidase (HOX) é a enzima limitante de velocidade no processo de degradação do heme, que pode degradar por oxidação a molécula do heme para produzir monóxido de carbono, ferro e biliverdina. A heme oxidase tem duas isoenzimas, heme oxidase 1 (HOX-1) e heme oxidase 2 (HOX-2). A HOX-2 desempenha basicamente um papel regulatório sob condições fisiológicas normais, enquanto a HOX-2 pode proteger as células e os tecidos em stress oxidativo.^26^

A expressão de HOX-1 pode ser promovida por uma variedade de estímulos, incluindo a doxorrubicina^27^ e o acúmulo de heme intracelular. Estudos anteriores revelaram que a expressão de HO-1 é regulada principalmente no nível da transcrição, e identificou-se que vários elementos regulatórios têm um papel importante na regulação para cima da HOX-1, incluindo elementos de resposta metal (MREs), elementos de resposta ao stress (StREs), AP-1, e NF-B.^28^ Por outro lado, demonstrou-se que Bach1 tem um efeito repressor na expressão de HOX-1.^29^ No presente estudo, identificamos que a HOX-1 nos cardiomiócitos de H9c2 foi inicialmente regulada para baixo quando tratada com baixa concentração de doxorrubicina (1μM e 2 μM) nas células H9c2 e, em seguida significativamente regulada para cima com incubação com 5μM e 10μM de doxorrubicina. Nossa pesquisa indica que serão necessários mais estudos com foco na interação entre vários reguladores de e elementos de gene da HOX-1 em concentrações diferentes de doxorrubicina.

Apesar de apresentar padrão semelhante ao da expressão de mRNA com HOX-1, o nível de proteína da HOX-2 foi elevado progressivamente juntamente com o aumento de concentração de doxorrubicina de 1μM a 5μM, e baixou abruptamente abaixo do nível de linha de base quando tratado com 10μM de doxorrubicina. A diferença no perfil de alteração do nível de proteína entre HOX-1 e HOX-2 pode ser devida à diferença em seus mecanismos de regulação.^30,31^ Em ratos, a expressão de HO-2 é modulada por glicocorticoides, por meio do elemento de resposta do glicocorticoide (GRE).^32^ Até onde sabemos, este é o primeiro estudo sobre o efeito da doxorrubicina na expressão de HOX-2 em cardiomiócitos. Entretanto, o mecanismo de ação preciso entre a doxorrubicina e a regulação de HOX-2 ainda é uma questão crítica não resolvida.

### Limitações do estudo

Falta de resultados na detecção de atividades de enzimas em células H9c2 era considerado como a principal limitação de nosso estudo. Enquanto isso, o nível de stress oxidativo, de acordo com a alteração do metabolismo de heme não foi explorada, o que pode elucidar melhor a relação entre enzimas metabólicas do heme e cardiotoxicidade induzida por doxorrubicina. Mais estudos cobrindo esses aspectos são necessários para esclarecer o efeito da doxorrubicina na biossíntese e no metabolismo do heme.

## Conclusões

O aumento da expressão de ALAS1 pode ter um papel na elevação do nível do heme quando o cardiomiócito H9c2 for exposto à doxorrubicina. Embora as HOX tenham sido reguladas para cima em concentrações de doxorrubicina de moderada a alta, seus efeitos degradadores foram sufocados pela ativação da síntese do heme não controlada. Os mecanismos específicos para a perda de controle de feedback negativo na formação do heme em tratamento com doxorrubicina, e o papel possível das ALAS como alvo terapêutico contra a citotoxicidade por heme induzida por doxorrubicina deverá ser estudada em investigações futuras.
