# Supplementary material for: Os Efeitos da Doxorrubicina na Biossíntese e no Metabolismo do Heme em Cardiomiócitos
Source: Arq Bras Cardiol. 2021 Feb 19;116(2):315–22. [Article in Portuguese] doi: 10.36660/abc.20190437 (PMC7909973; doi:10.36660/abc.20190437)
Supplement: Translation

# Translation: Effects of Doxorubicin on Heme Biosynthesis and Metabolism in Cardiomyocyte

**Authors:** Zuoyan Wang, Junyi Gao, Haobo Teng, Jianjun Peng

**Affiliations:** 1 ChinaCapital Medical University Affiliated Beijing Shijitan Hospital, Beijing - China

**Keywords:** Doxorubicin, Biosynthesis, Heme, Myocites, Cardiac, Cardiotoxicity, ALAS

## Abstract

**Background:**

Doxorubicin is associated with cardiotoxicity and late cardiac morbidity. Heme is related to cellular oxidative stress. However, its specific regulation in cardiomyocytes under doxorubicin effects has not yet been documented.

**Objective:**

This study seeks to evaluate the changing profiles of rate-limiting enzymes in the heme metabolism pathway under the effect of doxorubicin.

**Methods:**

H9c2 cardiomyocytes were incubated with doxorubicin at different concentrations (1,2,5,10μM respectively). The real-time PCR and Western Blot were used to determine the mRNA and protein expression for four pivotal enzymes (ALAS1, ALAS2, HOX-1, and HOX-2) regulating cellular heme metabolism, as well as the levels of heme were detected by ELISA. p<0.01 was considered significant.

**Results:**

This study observed a dose-dependent changing pattern in heme levels in H9c2 cells with the highest level at the 5μM concentration for doxorubicin, which occurred synchronously with the highest upregulation level of ALAS1, as well as the degradative enzymes, HOX-1, and HOX-2 in mRNA and protein expression. By contrast, ALAS2, contrary to the increasing concentrations of doxorubicin, was found to be progressively down-regulated.

**Conclusion:**

The increase in ALAS1 expression may play a potential role in the heme level elevation when H9c2 cardiomyocyte was exposed to doxorubicin and may be a potential therapeutic target for doxorubicin-induced myocardial toxicity. (Arq Bras Cardiol. 2021; 116(2):315-322)

## Introduction

With the continuing progression of anti-tumor drugs and radiotherapy, the survival rate of patients with malignant tumors has been improved, and their survival-span is significantly prolonged. However, the widespread use of anti-tumor drugs is accompanied by an increase in cardiovascular adverse events, which affects patient survival and quality of life. Anthracyclines are drugs derived from streptomycin, including doxorubicin, and epirubicin, which are widely used to treat breast cancer, small cell lung cancer, myeloma, sarcoma, lymphoma, and leukemia. Myocardiopathy and subsequent heart failure are the most serious manifestation of cardiotoxicity caused by anthracycline drugs in chemotherapy. Moreover, the cardiovascular toxicity of anthracyclines is dose-dependent and irreversible.^1,2^

The exact mechanism of anthracycline drugs that induced myocardial toxicity remains unclear, though a variety of theories have been proposed, including the inhibition of DNA replication and RNA transcription, DNA damage caused by free radicals, lipid peroxidation and alkylation, DNA cross-linking, interference with DNA unwinding, inhibition of topoisomerase Ⅱ, among others.

Heme is an important porphyrin ligand, as a supplement of the heme protein, and is responsible for the physiological function of O_2_ transport in the body. Many biological functions related to life, such as electron transport, oxygen storage, signal transduction, and gene expression are controlled by different heme proteins. Recent studies found that heme levels increased significantly in rat models of myocardial ischemic heart failure, suggesting that heme may play an important role in ischemic and hypoxic myocardial injury.^3,4^ However, there has been no published literature that specifically explored the changing of heme biosynthesis or metabolism in cardiomyocyte under anthracyclines treatment.

Heme biosynthesis is initiated by the formation of 5-aminolevulinic acid (ALA) from glycine (Gly) and succinyl-CoA, catalyzed by ALA synthase (ALAS), which has two isoenzymes: ALAS1 and ALAS2. On the other hand, heme oxygenase (HOX) mediates the first step of heme catabolism, and it cleaves heme to form biliverdin. Two active isoforms of HOX have been identified: the inducible HOX-1 and the less regulated HOX-2.

The purpose of this study was to elucidate the changing profiles of four pivotal enzymes, aminolaevulinic acid synthase 1 (ALAS1), aminolaevulinic acid synthase 2 (ALAS2), heme oxygenase 1 (HOX-1), and heme oxygenase 2 (HOX-2), in H9c2 cardiomyocyte under doxorubicin treatment.

## Methods

### Cell culture and doxorubicin treatment

H9c2 cardiomyocytes were purchased from American Type Culture Collection (ATCC, Manassas, VA)and then cultured with DMEM/F12 medium containing 10% fetal bovine serum and 1% double-antibody medium, under the conditions of 37°C and 5% CO_2_ in a humidified chamber. The H9c2 cells were then plated onto 6-well plates at 2 × 10^5^ cells/well in a volume of 2 mL and cultured in DMEM/F12 medium containing 10% fetal bovine serum for 24 h. After, different doses of doxorubicin (1,2,5,10 μM, respectively) were added to the plate well. The doxorubicin-untreated cells (treated with saline) represented the control group. After a 24h-incubation at 37ºC, the cells of different wells were harvested separately by centrifugation (10,000 rpm for 10 minutes at 4°C) and used for further study below.

### Quantitative real-time PCR

Total RNA was extracted from H9c2 cells of all the groups, using TRIzol (Invitrogen; Thermo Fisher Scientific, Inc., Waltham, MA, USA) and reverse transcribed with the SuperScript Double-Strand Synthesis kit (Invitrogen; Thermo Fisher Scientific, Inc.), according to the manufacturer’s instructions, and amplified on a 7500 Fast real-time PCR system with SYBR GreenER qPCR SuperMix Universal (Invitrogen; Thermo Fisher Scientific, Inc., Waltham, MA, USA). Primers used for amplification of ALAS1, ALAS2, HOX-1, and HOX-2 were designed using Primer3 (v. 0.4.0) software. The following primers were used for real-time PCR:

ALAS1-F:TTGCCAAAGTCCGTTTCC

R: TGTAGTCATCTGCCATAGGG 3’;

ALAS2-F: TCAAGGGAGAGGAGGGTCAAG

R: ACGAGGCACAGTTGGGTAG

HOX-1-F:TCGACAACCCCACCAAGTT

R:CTGGCGAAGAAACTCTGTCT

HOX-2-F:GCTTACACTCGTTACATGGG

R: CACATGCTCGAACAGGTAGA

GAPDH-F: GATGACATCAAGAAGGTGGTGA

R:ACCCTGTTGCTGTAGCCATATTC.

The reaction conditions were as follows: 95°C for 3 min; 95°C for 30 sec, 55°C for 20sec, and 72°C for 20sec with 40 cycles. Melting curve analyses were performed to verify their amplification specificity. The expression values of all targeted genes from each sample were calculated by normalizing with internal control GAPDH and calculated using the 2-ΔΔCT method.

### Western Blot

H9c2 cells were trypsinized, centrifuged for 5 min at 1,000 x g and washed with cold PBS. The H9c2 cells were then resuspended in lysis buffer with protease and phosphatase inhibitors. The cell lysate was kept in ice and vortexed. After centrifugation for 20 min at 13,000×g, the supernatant was separated and stored at −80 °C until use. After denaturation, 20μg of total protein were loaded onto 12% sodium dodecyl sulfate-polyacrylamide gel electrophoresis (SDS-PAGE) gels for protein separation, and electrophoretically transferred to a polyvinylidene difluoride (PVDF) membrane. After blocking with 5%milk in TBST (50mM Tris-HCl, pH 8;154 mM NaCl and 0.1% tween20) for 2 hours at room temperature, the membranes were probed with specific antibodies against ALAS1 (dilution,1:1,000; rabbit polyclonal; cat. no. 16200-1-AP; Proteintech), ALAS2 (dilution,1:1,000; rabbit polyclonal; cat. no. ab136799; Abcam), HOX-1 (dilution,1:1,000; rabbit polyclonal; cat. no. ab230513; Abcam), HOX-2 (dilution,1:500; rabbit polyclonal; cat. no. ab229960; Abcam), and GAPDH (dilution, 1:1,000; clone 6C5; cat. no. ab8245; Abcam) overnight at 4°C. Horseradish peroxidase (HRP)-labeled secondary antibodies (1:1,000 dilution; cat.nos. A0208, A0216; Beyotime) was added, followed by incubation at room temperature for 2 h. Membranes were visualized by enhanced chemiluminescence kits (Biorbyt, Ltd., Cambridge, UK). To quantify the targeting protein expression, the X-ray films were scanned, and band intensities were quantified by ImageJ 1.47 software. The results were normalized to GAPDH.

### Intracellular heme measurement

Enzyme-linked immune sorbent assay (ELISA) was employed to determine intracellular heme levels. H9c2 cells of each group were lysed and followed by centrifugation at 12,000 rpm for 15 minutes at 4°C to remove debris. The protein concentration of the cell lysate was quantified by an enhanced BCA Protein Assay kit (Beyotime). The heme in H9c2 cell lysates was measured by using QuantiChrom Heme Assay (BioAssay Systems) following the manufacture’s protocol and normalized to a protein concentration of each sample.

### Flow cytometry to determine the apoptosis rate in H9c2 cells

Apoptosis was assessed using the Annexin V-FITC/PI Apoptosis Detection kit (KeyGEN, China) according to the manufacturer’s protocol. After the intervention, approximately 1×10^5^ H9c2 cells of each group were washed and digested with trypsin, and then resuspended with 1x PBS (4°C) and centrifuged at 2,000 rpm for 5-10 minutes to wash the cells. The cells were resuspended in 500μl of the buffer, followed by the addition of 5 μl of Annexin V-FITC and 5 μl of PI. The cells were incubated in the dark for 15 minutes at room temperature. Next, the cells of each group were examined for apoptosis rate by flow cytometry (BD Accuri™ C6). The experiment was repeated three times. The apoptotic rate was quantified as the percentage of cells stained with Annexin V.

### Statistical Analysis

Data in the present study are normally distributed, which was verified by Shapiro- Wilk test, and are expressed as mean ± SD. One-way ANOVA, followed by Tukey’s post hoc test, was used to examine the statistical significance of differences between groups. Statistical analysis was performed by using the SPSS 24.0 Statistical Package Program for Windows (SPSS Inc., Chicago, IL, USA). A two-sided p-value of <0.01 was considered significant.

## Results

### The changing of the heme level in H9c2 cells with a different concentration of doxorubicin

As shown in Figure 1, compared with the control group (5088.4±153.1ng/ml), heme levels of H9c2 cells were significantly up-regulated to a 1.27-fold (6493.1±138.8 ng/ml), a 1.56-fold (7498.9±110.2ng/ml), and a maximum 2.34-fold (11896.6±187.3ng/ml) increase in 1μM, 2μM, and 5μM doxorubicin groups, separately (p<0.01). This up-regulation trend was dropped back to a 1.95-fold (9911.9±286.8ng/ml) increase in the 10μM doxorubicin group, as compared to the control group (p<0.01).

**Figure 1:** Effects of doxorubicin on heme levels in H9c2 cells exposed to saline (control group) or doxorubicin with different concentrations for 24 hours. Heme levels were measured by ELISA. Data are presented as the mean±standard deviation. *p<0.01, compared with the control group.

### The Effects of doxorubicin on the apoptosis rate of H9c2 cells

The flow cytometry analysis showed that, compared with the control group treated with saline, the apoptosis rate of H9c2 cells treated with different concentrations of doxorubicin was significantly increased, as shown in Figure 2. When incubated with 1, 2, 5, and 10μM doxorubicin for 24h, the total apoptosis rate, including both early and end-stage apoptosis, of H9c2 was increased to 10.6±1.6%, 41.1±1.9%, 60.5±3.6%, and 76.0±2.5%, respectively, as compared to 2.1±0.5% in the control group (p<0.01).

**Figure 2:** – Flow cytometry analysis to evaluate the effects of doxorubicin on H9c2 cell viability. H9c2 cells were pretreated with saline (control) and doxorubicin at 1, 2, 5 and 10μM, respectively, for 24h. (A) Representative flow cytometry analyses of five individual experiments corresponding to control and different concentration doxorubicin treatment, respectively. (B) Statistical graph of annexin V-FITC/PI staining. Results was expressed as mean±SD. * p<0.01 vs control group.

### Regulation of aminolaevulinic acid synthase 1 (ALAS1) and aminolaevulinic acid synthase 2 (ALAS2) messenger RNA (mRNA) expression in H9c2 cells after doxorubicin treatment

As the first step and rate-limiting enzymes for heme synthesis that occur in the mitochondria, the mRNA expression of ALAS1 and ALAS2 under doxorubicin treatment were evaluated. After incubating with 1μM and 2μM doxorubicin, the mRNA expressions of ALAS1 were down-regulated (though not statistically significant). After treating with 5μM and 10μM doxorubicin, the ALAS1 mRNA expressions were statistically increased to 41.1-fold and 375.3-fold, separately, as compared to the control group. Unlike ALAS1, ALAS2 mRNA expression proved to be progressively and significantly down-regulated (1μM group:0.88-fold, 2μM group: 0.83-fold, 5μM group: 0.49-fold, 10μM group:0.31-fold respectively, as shown in Figure 3A.)

**Figure 3:** – (A) mRNA expression changes of ALAS1 and ALAS2 under doxorubicin treatment. ALAS1, aminolaevulinic acid synthase 1; ALAS2, aminolaevulinic acid synthase 2. (B) Characteristics of mRNA expression changes for heme catabolic enzymes under doxorubicin treatment. HOX-1, heme oxygenase 1; HOX-2, heme oxygenase 2. * indicate p<0.01 vs. Control group.

### Regulation of mRNA expression for HOX-1 and HOX-2 in H9c2 cells after doxorubicin treatment

The cytoplastic rate-limiting enzymes in heme catabolism, HOX-1 and HOX-2, were examined. It was found that after having been incubated with an increasing level of doxorubicin, the HOX-1 and HOX-2 mRNA exhibited the same regulation pattern, although with different changing levels. No significant change was observed in mRNA expression after 1μM and 2μM doxorubicin treatment, when compared with the control group, for either HOX-1 or HOX-2. After incubating with 5μM doxorubicin, the mRNA expression for HOX-1 and HOX-2 significantly up-regulated to 4.3-fold and 15.5-fold changes, respectively (p<0.01). But after treatment with 10μM doxorubicin, the up-regulation was declined to the extent of 2.6-fold and 3.2-fold, separately, when compared with the control group (p<0.01), as shown in Figure 3B.

### Regulation of ALAS1, ALAS2, HOX-1, and HOX-2 protein levels after doxorubicin treatment

Western blot analysis showed that, identical to their mRNA expression pattern, ALAS1 protein levels were significantly down-regulated in the 2μM doxorubicin group and up-regulated in 10μM doxorubicin groups, when compared with the control group. ALAS2 protein levels were found to be progressively down-regulated as the doxorubicin concentration increased from 1μM to 10μM, as shown in Figure 4A.

**Figure 4:** – Analysis data of western blotting for ALAS (A) and HOX (B) protein level change in H9c2 cells at different concentrations for doxorubicin. ALAS1, aminolaevulinic acid synthase 1; ALAS2, aminolaevulinic acid synthase; HOX-1, heme oxygenase 1; HOX-2, heme oxygenase 2. Each western blot was conducted three times. * indicate p<0.01 vs. control group.

Protein expression of HOX-1 was slightly down-regulated under 1μM and 2μM doxorubicin treatment, and then sharply up-regulated when compared with the control group. However, inconsistent with the mRNA expression pattern, our study detected a progressive increase in the HOX-2 protein level, with an increase in the doxorubicin concentration from 1μM to 5μM, and then a decline to the baseline level when treated with 10μM doxorubicin (Figure 4B).

## Discussion

Doxorubicin is a kind of anthracycline drug, which is an effective broad-spectrum anti-tumor drug. It is widely used in the treatment of various malignant tumors, such as breast cancer, lung cancer, and lymphoma.^5^ But the toxic cardiac effects of doxorubicin in a clinical chemotherapy dose is severe and dose-dependent, which can cause cardiomyopathy and congestive heart failure, and thus greatly limits its clinical use.^6^ Different from the mechanism of its anti-tumor activity, the primary mechanism of cardiac toxicity induced by doxorubicin, is the generation of iron-mediated reactive oxygen species (ROS) and the subsequent promotion of myocardial oxidative stress.^7^ Heme is an essential mediator of the biochemical availability of iron.^8^The function of the heme molecule varies according to the binding protein it coordinates with. It may serve as a mediator of oxygen transport and storage in hemoglobin^9^ or myoglobin,^10^ whereas it acts as an electron transporter in cytochromes and is the critical source of redox-active iro.^11^ Bhoite-Solomon et al. found that free heme is toxic to the myocardium and caused cytolysis through sarcolemma damage in a concentration-dependent manner.^12^ In addition to cardiomyocyte, free heme can also be toxic to human epithelial cells and neuron-like cells^13,14^ through oxidative stress caused by cell apoptosis or necrosis. Moreover, free heme can cause endothelial cell injury by stimulating the expression of inflammatory factors.^15,16^

However, unlike the abundant studies in the iron-mediated oxidative injury in cardiomyocyte treated with anthracycline drugs, the intracellular heme level variation and the regulation processes of its synthesis and metabolism have not been well evaluated in cardiomyocyte under anthracycline treatment. The present study systematically examined the variation characteristics of biosynthetic and degradative enzymes for heme for the first time.

In the heme biosynthesis pathway, there are eight enzymes involved, among which four are mitochondrial enzymes, and four are cytoplasmic enzymes.^17^ For the first step of heme synthesis, glycine and succinyl coenzyme A are condensed into δ-aminolevulinic acid (ALA). This reaction needs to be catalyzed by aminolevulinic acid synthase (ALAS).^18^ There are two types of ALAS: ALAS1 and ALAS2. The former has a universal expression, while the latter is dominantly expressed in red blood cell precursor cells.^19^ The present study found that not only ALAS1, but also ALAS2, were both expressed in the H9c2 cells at baseline levels. After the treatment with doxorubicin, ALAS1 and ALAS2 mRNA expression exhibit two distinct changing patterns. The ALAS1 mRNA and protein expression at first presented a trend of inhibition (though not statistically significant in our study) with 1μM and 2μM doxorubicin and then showed a dramatic up-regulation pattern with 5μM and 10μM doxorubicin treatment.

By contrast, mRNA and protein expression of ALAS2 were progressively suppressed to the lowest level when treated with 10μM doxorubicin, with 69.0% and 35.0% reductions, respectively. This phenomenon indicates that, under the effect of doxorubicin, the regulation of ALAS1 and ALAS2 may occur through the different pathways and seek different targeting bioprocesses. The step-down-regulation of ALAS2 can be explained by the negative feedback from a progressive elevation of heme level. This effect might be achieved by repressing ALAS2 mRNA transcription, as shown in our real-time PCR results, as well as by interrupting ALAS2 formation through a block of the ALAS2 precursor into mitochondria.^20,21^ In red blood cells, the expression of ALAS2 is determined by the trans-activation of nuclear factors GATA-1, CACC box, and NF-E2-binding sites in the promoter area, and its synthesis is regulated by the amount of free iron. ^22^ However, the regulation mechanism of ALAS2 in cardiomyocyte under doxorubicin treatment will need to be further researched.

In our study, ALAS1, which was supposed to be strictly under negative feedback control at a low level to prevent a high cytotoxic heme level, was overexpressed both in gene and protein aspects, despite the simultaneously existing high heme level. Podvinec et al.^23^ reported that drugs that induce cytochromes P450 and other drug-metabolizing related enzymes might simultaneously and transcriptionally up-regulate ALAS1 expression, which is the first step in the heme synthesis, to coordinate with the need of up-regulated cytochrome P450 activity. This process was mediated through two enhancer elements, located 20 and 16 kb upstream of the transcriptional start site of ALAS1, and their interaction with the xenoreceptors NR1I2 and NR1I3.^23^ Zordoky et al.^24^ revealed that doxorubicin caused a significant induction of several cytochrome P450 genes, such as CYP1A1, CYP1A2, CYP1B1, and CYP2B2 gene expression in a concentration-dependent manner. NR1I2 and NR1I3 have also been proved to be the crucial nuclear receptors mediating the interaction between doxorubicin and metabolizing enzymes.^25^ The precise mechanism for the regulation of ALAS expression patterns in the cardiomyocyte will need to be elucidated in future study.

Heme oxidase (HOX) is the rate-limiting enzyme in the process of heme degradation, which can oxidatively degrade the heme molecule to produce carbon monoxide, iron, and biliverdin. Heme oxidase has two isoenzymes, heme oxidase 1 (HOX-1) and heme oxidase 2 (HOX-2). HOX-2 mainly plays a regulatory role under normal physiological conditions, while HOX-2 may protect cells and tissues under oxidative stress.^26^

The HOX-1 expression may be promoted by a variety of stimuli including doxorubicin^27^ and intracellular accumulation of heme. Previous studies revealed that HO-1 expression is primarily regulated at the transcriptional level, several regulatory elements have been identified to play important roles in up-regulating HOX-1, including metal response elements (MREs), stress response elements (StREs), AP-1, and NF-B. ^28^ On the other hand, Bach1 has proven to have a repressor effect on HOX-1 expression.^29^The present study found HOX-1 in H9c2 cardiomyocytes was down-regulated in the first place when treated with low concentration doxorubicin (1μM and 2μM) in H9c2 cells and then significantly up-regulated with 5μM and 10μM doxorubicin incubation. Our research indicates that further studies focusing on the interaction between various regulators and HOX-1 gene elements under different doxorubicin concentration will be needed.

In spite of exhibiting a similar pattern of mRNA expression with HOX-1, the HOX-2 protein level was progressively elevated, along with the doxorubicin concentration, which increased from 1μM to 5μM and abruptly declined below the baseline level when treated with 10μM doxorubicin. The difference in the changing profile of the protein level between HOX-1 and HOX-2 may be due to the difference in their regulation mechanisms.^30,31^In rats, HOX-2 expression is modulated by glucocorticoids through the glucocorticoid responsive element (GRE).^32^ To the best of our knowledge, this is the first study on the effect of doxorubicin on HOX-2 expression in cardiomyocytes; however, the precise mechanism of action between doxorubicin and HOX-2 regulation remains a critical, unsolved question.

### Study limitations

The lack of results in detecting enzyme activities in H9c2 cells was believed to be the main limitation of our study. Meanwhile, the oxidative stress level, according to the changes in heme metabolism, has not been explored, which may well elucidate the relationship between heme metabolic enzymes and doxorubicin-induced cardiotoxicity. Further studies covering these aspects are warranted to clarify the effect of doxorubicin on the heme biosynthesis and metabolism.

## Conclusions

The increase in ALAS1 expression may play a potential role in the heme level elevation when H9c2 cardiomyocyte is exposed to doxorubicin. Although HOXs were upregulated under moderate-high concentration doxorubicin treatment, their degradative effects were overwhelmed by the uncontrolled heme synthesis activation. The specific mechanisms for the loss of negative feedback control to heme formation under doxorubicin treatment and the potential role of ALAS as a therapeutic target against the doxorubicin-induced heme cytotoxicity will need to be studied in future investigations.
